# Patient-reported cognitive function before and after glioma surgery

**DOI:** 10.1007/s00701-022-05261-3

**Published:** 2022-06-06

**Authors:** Stine Schei, Ole Solheim, Øyvind Salvesen, Tor Ivar Hansen, Lisa Millgård Sagberg

**Affiliations:** 1 Department of Public Health and Nursing, Norwegian University of Science and Technology, Trondheim, Norway; 2 Department of Neurology, St. Olavs Hospital, Trondheim, Norway; 3 Department of Neuromedicine and Movement Science, Norwegian University of Science and Technology, Trondheim, Norway; 4 Department of Neurosurgery, St. Olavs Hospital, Trondheim, Norway; 5 Unit for Applied Clinical Research, Department of Clinical and Molecular Medicine, Norwegian University of Science and Technology, Trondheim, Norway

**Keywords:** Brain neoplasms, Cognition, Glioma, Patient-reported outcome measures, Neurosurgery

## Abstract

**Background:**

Little is known about the extent to which glioma patients experience subjective changes in cognitive function following surgery. We sought to assess patient-reported cognitive function before and after glioma surgery and explore potential factors associated with cognitive change.

**Methods:**

In a prospective population-based study, patient-reported cognitive function was measured in 182 patients undergoing primary surgery for diffuse glioma (141 high-grade gliomas (HGG) and 41 low-grade gliomas (LGG)) by using the European Organisation for Research and Treatment of Cancer (EORTC) QLQ-C30 cognitive function subscale preoperatively and at 1 and 6 months postoperatively. Binomial logistic regression models were used to assess factors possibly associated with patient-reported cognitive changes.

**Results:**

In the HGG group, the mean cognitive function score increased from 70.9 (95% 66.6, 75.2) preoperatively to 85.1 (95% CI 81.2, 89.0) (*p* < 0.001) and 83.3 (95% CI 79.1, 87.6) (*p* < 0.001) at 1 and 6 months postoperatively, respectively. In the LGG group, the mean score was 80.9 (95% CI 74.4, 87.4) preoperatively and remained stable at postoperative follow-ups. Females reported lower scores than males. At an individual level, both improvement and deterioration in cognitive scores were frequently seen in LGG and HGG patients after surgery. Preoperative use of corticosteroids and large tumor volume were predictors for cognitive improvement at 1 month postoperatively. No predictors were identified for cognitive improvement at 6 months and worsening at 1 and 6 months.

**Conclusion:**

Many glioma patients experience perioperative subjective changes in cognitive function after surgery. At group level, HGG patients reported improved cognitive function after surgery, while LGG patients reported stable cognitive function. Preoperative use of corticosteroids and large tumor volume were independently associated with postoperative improvement.

## Introduction

Cognitive impairment is common and adds to the symptom burden of diffuse glioma. Previous studies suggest that up to 50–60% of diffuse glioma patients exhibit subjective and objective impairments before surgical treatment [34, 41], which may have detrimental effects on both quality of life and survival [4, 22, 42].

The effects of surgery on cognitive function in low-grade glioma (LGG) and high-grade glioma (HGG) have been assessed with objective tests in several neuropsychological studies [11, 18, 39, 40]. A meta-analysis concluded that surgery seems to have an overall beneficial effect on most cognitive functions in diffuse glioma patients [28], but the result is debated as it likely reflects an overestimation of postoperative cognitive performance [33]. However, the patients’ perspective is also relevant and might differ from the results of objective tests [8, 15, 31]. Unlike objective tests, patient-reported cognitive function reflects the patients’ self-perceived function, which may be of value for assessing the net clinical effect of treatment [3].

So far, little is known about the impact of surgery on patients’ self-perceived cognitive function. In a previous study of quality of life in surgical glioma patients, the patients reported both improvement and worsening after surgery [45]. However, this was a small pilot study with only 22 patients. More knowledge about patient-reported cognitive function may be useful when informing patients on what to expect after their operation.

This longitudinal study aimed to assess perioperative and postoperative changes in patient-reported cognitive function in patients undergoing primary resections for diffuse glioma and explore possibly associated factors.

## Material and methods

### Study population and design

All adult patients (≥ 18 years) undergoing primary surgical resection under general anesthesia for diffuse glioma at the neurosurgical department at St. Olavs Hospital (Trondheim, Norway) from September 2011 to December 2019 were screened for inclusion. The neurosurgical department serves about 750,000 inhabitants within a defined geographical catchment region, ensuring population-based referral. The patients were identified from a prospective regional brain tumor surgery registry. We only included patients with a histopathologically confirmed diffuse grade II–IV glioma according to the 2007 or 2016 World Health Organization classifications [23, 24]. Exclusion criteria were known dementia and operations done in more than one setting (e.g., multifocal resections).

### Study variables and data collection

We assessed patient-reported cognitive function with the Norwegian version of the European Organisation for Research and Treatment of Cancer (EORTC) QLQ-C30 questionnaire cognitive functioning subscale [1]. This subscale contains two questions about memory and concentration, and the questions are answered on a four-point scale from “not at all” to “very much.” The patients completed the EORTC QLQ-C30 questionnaire 1–3 days before surgery by themselves or with practical assistance from a nurse or family member, if needed. A trained study nurse collected follow-up assessments through structured telephone interviews after approximately 1 (median 33 days; range 23–63 days) and 6 months (median 184 days; range 144–211 days).

The Karnofsky performance status (KPS) score was prospectively rated preoperatively by the operating surgeon and postoperatively by a study nurse based on the information from the telephone interviews [27]. In patients with missing prospective KPS (*n* = 10), a retrospective estimation was done based on medical journals to classify the patients as functionally dependent (< 70) or independent (≥ 70). Other patient and treatment characteristics were obtained from electronic medical journals. Charlson comorbidity index was used to classify comorbidity [9], and postoperative complications within 30 days were categorized according to Landriel classification system [19]. Magnetic resonance images (MRI) were routinely acquired < 72 h before surgery and ≤ 48 h after surgery. The tumor volumes were estimated by semi-automatic tumor segmentation of MRI images using the software packages 3D Slicer version 4.3.1–4.11 (3D Slicer, Boston, Massachusetts) [13] and BrainVoyager version 1.2 (Brain Innovation, Maastricht, Netherlands). Preoperative tumor volume in contrast-enhancing tumors was defined as necrotic tissue plus contrast-enhancing border seen on T1 MRI scans. In tumors without contrast enhancement, the T2/FLAIR volume was used. Lateralization was categorized according to the center of mass in each tumor, while multifocal bilateral tumors were classified as a separate group. Location was categorized based on which lobe that was involved, whereas tumors in several lobes were organized into a separate group. Tumor progression within 6 months of follow-up was determined according to the Response Assessment in Neuro-Oncology (RANO) criteria [44].

### Statistical analysis

Statistical analyses were conducted in SPSS version 27.0 (IBM Corp., Armonk, NY, USA). Data have been summarized as mean, medians, or frequencies, as appropriate. Answers from the cognitive items were transformed into a 0–100 scale according to the EORTC scoring manual, where a higher score represents better cognitive functioning [12]. We assessed changes in patient-reported cognitive function at both group level and individual patient level. *T* tests were performed to analyze longitudinal changes within groups from preoperative to 1 and 6 months of follow-up. To determine the proportion with cognitive change at individual patient level, the previously published minimal clinically important difference (MCID) of ± 10 was applied to categorize changes into “improvement,” “unchanged,” and “deterioration” [25]. In addition to the observed data, we have also presented estimated scores at group level in an attempt to adjust for potential bias due to missing data. Patients who died before follow-up were excluded from the estimations. In total, 170 patients were alive at all assessment points, two patients were dead at 1 month, and ten patients died between 1 and 6 months of follow-up (three scenarios). For each of the three scenarios, mixed binomial regression model was used to estimate the mean cognitive function score at relevant assessment points. Further, the three binomial mixed models were combined using the observed proportions of the three scenarios. Time was used as the fixed effect and person ID as the random effect.

Variables possibly associated with changes in patient-reported cognitive function were explored by binomial logistic regression analyses. Variables significantly associated with changes in the univariable analyses were included in the multivariable models. The statistical significance level was set at *p* ≤ 0.05. Variables with < 15 cases were excluded. The concordance index was used to measure the model’s predictive accuracy, and the Nagelkerke *R* square value was used to assess how much variation in cognitive function the model explained.

### Ethics

This study was approved by the Regional Committee for Medical and Health Research Ethics in South and East Norway (reference number 67005). All patients in the glioma population provided written informed consent as part of another project (reference number 2011/974) or in the Health Region Mid-Norway Brain Tumor Registry (reference number 2015/215), and the data collection adhered to the guidelines of the Helsinki Declaration.

## Results

**Fig. 1:**
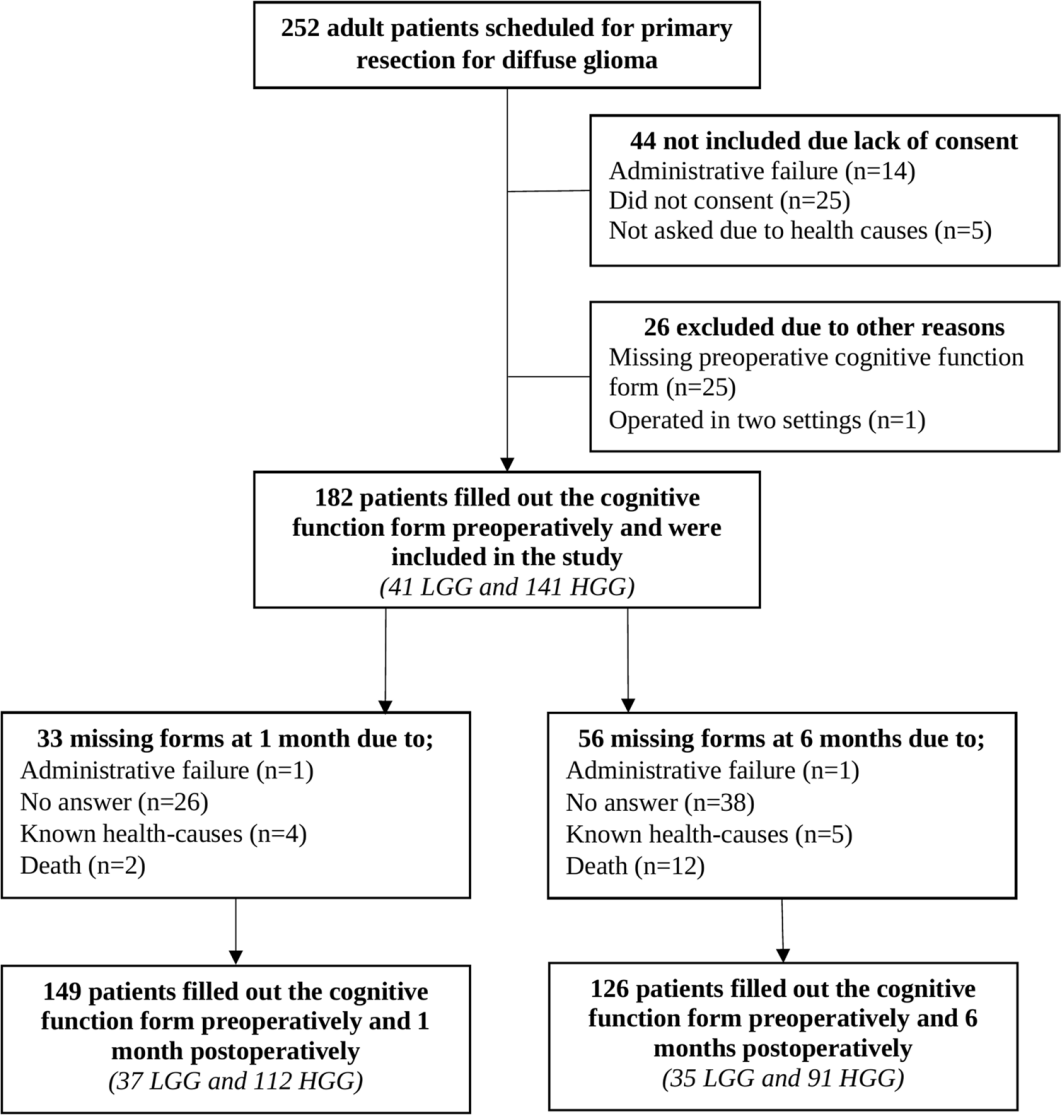
The inclusion process. *LGG*, low-grade glioma; *HGG*, high-grade glioma

Patient-reported cognitive function data were available at preoperative baseline in 182 of 252 (72%) patients, of whom 149 (82%) and 126 (69%) completed the questionnaire at 1 and 6 months after surgery, respectively. The inclusion process is presented in Fig. 1.

### Patients and treatment characteristics

Preoperative baseline and postoperative treatment and disease characteristics in both LGG and HGG patients are presented in Table 1. The median age was 39 years (range 18–69 years) in LGG patients and 61 years (range 28–80 years) in HGG patients. Preoperatively, more HGG were functionally dependent (11% vs. 2%), had larger tumor volumes (median 26.3 ml vs. 11.8 ml), and were more often treated with corticosteroids than patients with LGG (80% vs. 10%). Tumor location in multiple lobes (36%) was more common in HGG, while frontal location (58%) was more common in patients with LGG. Postoperatively, HGG patients more often received oncological treatment, and tumor progression within 6 months follow-up was seen in 48% of the HGG patients and 7% of the LGG patients.

**Table 1:** Baseline and postoperative treatment and disease characteristics

	Low-grade glioma	High-grade glioma
	*n* = 41	*n* = 141
Baseline characteristics		
Age in years, *median* (range)	39 (18–69)	61 (28–80)
Female sex, *n* (%)	18 (44)	48 (34)
IDH status, *n* (%)		
IDH-mutant	35 (86)	13 (9)
IDH-wild-type	5 (12)	112 (80)
Missing	1 (2)	16 (11)
Tumor lateralization, *n* (%)		
Right	23 (56)	66 (47)
Left	18 (44)	72 (51)
Bilateral	0 (0)	3 (2)
Tumor location, *n* (%)		
Frontal	24 (58)	43 (30)
Temporal	3 (7)	38 (27)
Parietal	2 (5)	7 (5)
Occipital	0 (0)	2 (1)
Cerebellum / brainstem	2 (5)	0 (0)
Deep cerebral^a^	4 (10)	1 (1)
Multiple lobes	6 (15)	50 (36)
Preoperative Karnofsky performance status score, *n* (%)		
≥ 70	40 (98)	126 (89)
< 70	1 (2)	15 (11)
Charlson comorbidity index ≥ 2, *n* (%)	2 (5)	4 (3)
Preoperative tumor volume ml, *median* (range)^b^	11.8 (0.75–163.1)	26.3 (0.96–200.9)
Preoperative use of corticosteroids, *n* (%)	4 (10)	113 (80)
Preoperative use of antiepileptic drugs, *n* (%)	14 (34)	45 (32)
Treatment and disease characteristics after resection		
Extent of resection, *n* (%)^c^		
Gross total (100%)	15 (37)	41 (30)
Subtotal (< 100%)	25 (63)	97 (70)
Landriel grade II–IV complications within 30 days, *n* (%)	6 (15)	17 (12)
Postoperative radiotherapy and/or chemotherapy within, *n* (%)		
1 month follow-up	0 (0)	120 (85)
6 months follow-up	3 (7)	139 (99)
Tumor progression within 6 months follow-up, *n* (%)^d^	3 (7)	65 (48)

*IDH*, isocitrate dehydrogenase

^a^Basal ganglia/thalamus/corpus callosum/insula

^b^*N* = 180 due to missing MRI

^c^*N* = 178 due to missing MRI

^d^*N* = 176 due to missing MRI

### Changes in cognitive function at group level

Observed and estimated patient-reported cognitive function scores in LGG and HGG patients at each assessment point are presented in Table 2. Preoperatively, the mean EORTC cognitive function score was 80.9 (95% CI 74.4, 87.4; SD ± 20.6) in the LGG group and 70.9 (95% CI 66.6, 75.2; SD ± 26) in the HGG group. In the LGG group, the score remained stable after surgery. However, in the HGG group, the mean score significantly improved to 85.1 (*p* < 0.001) at 1 month and 83.3 (*p* < 0.001) at 6 months of follow-up. There were only minor differences in the mean scores when comparing observed and estimated data. In a post hoc analysis of patient-reported cognitive function score in patients with available IDH status, we found similar results. The mean preoperative cognitive function score was 78.1 (95% CI 71.2, 85.0) in IDH-mutant gliomas and 71.6 (95% CI 67.0, 76.3) in IDH-wild-type gliomas.

**Table 2:** Mean (95% CI) EORTC cognitive function score preoperatively and at 1 and 6 months postoperative follow-up in low-grade and high-grade glioma

	Observed values				Estimated values	
	Low-grade glioma		High-grade glioma		Low-grade glioma	High-grade glioma
	Mean (95% CI)	*p* value*	Mean (95% CI)	*p* value*	Mean (95% CI)	Mean (95% CI)
Preoperative	80.9 (74.4, 87.4)	-	70.9 (66.6, 75.2)	-	81.0 (72.9, 87.2)	70.8 (66.2, 75.0)
1 month	81.1 (74.1, 88.0)	0.968	85.1 (81.2, 89.0)	< 0.001	80.7 (72.3, 87.1)	84.0 (80.1, 87.4)
6 months	81.4 (73.0, 89.8)	0.919	83.3 (79.1, 87.6)	< 0.001	81.2 (72.8, 87.6)	81.3 (76.7, 85.2)

*CI*, confidence Interval

^*^Changes from preoperative baseline

When comparing patient-reported cognitive function between sex, female patients had lower cognitive function scores than male patients (Table 3).The mean preoperative cognitive function score was 65.1 (95% CI 57.8, 72.5) in females and 77.7 (95% CI 73.9, 81.6) in males. Both sexes significantly improved at 1 and 6 months of follow-up.

**Table 3:** Mean (95% CI) EORTC cognitive function score preoperatively and at 1 and 6 months postoperative follow-up in females and males

	Sex			
	Females		Males	
	Mean (95% CI)	*p* value*	Mean (95% CI)	*p* value*
**Preoperative**	65.1 (57.8, 72.5)	-	77.7 (73.9, 81.6)	-
**1 month**	81.7 (75.4, 88.0)	< 0.001	85.4 (81.4, 89.4)	0.007
**6 months**	77.0 (69.9, 84.0)	0.022	85.7 (81.3, 90.1)	0.007

*CI*, confidence interval

^*^Changes from preoperative baseline

**Fig. 2:**
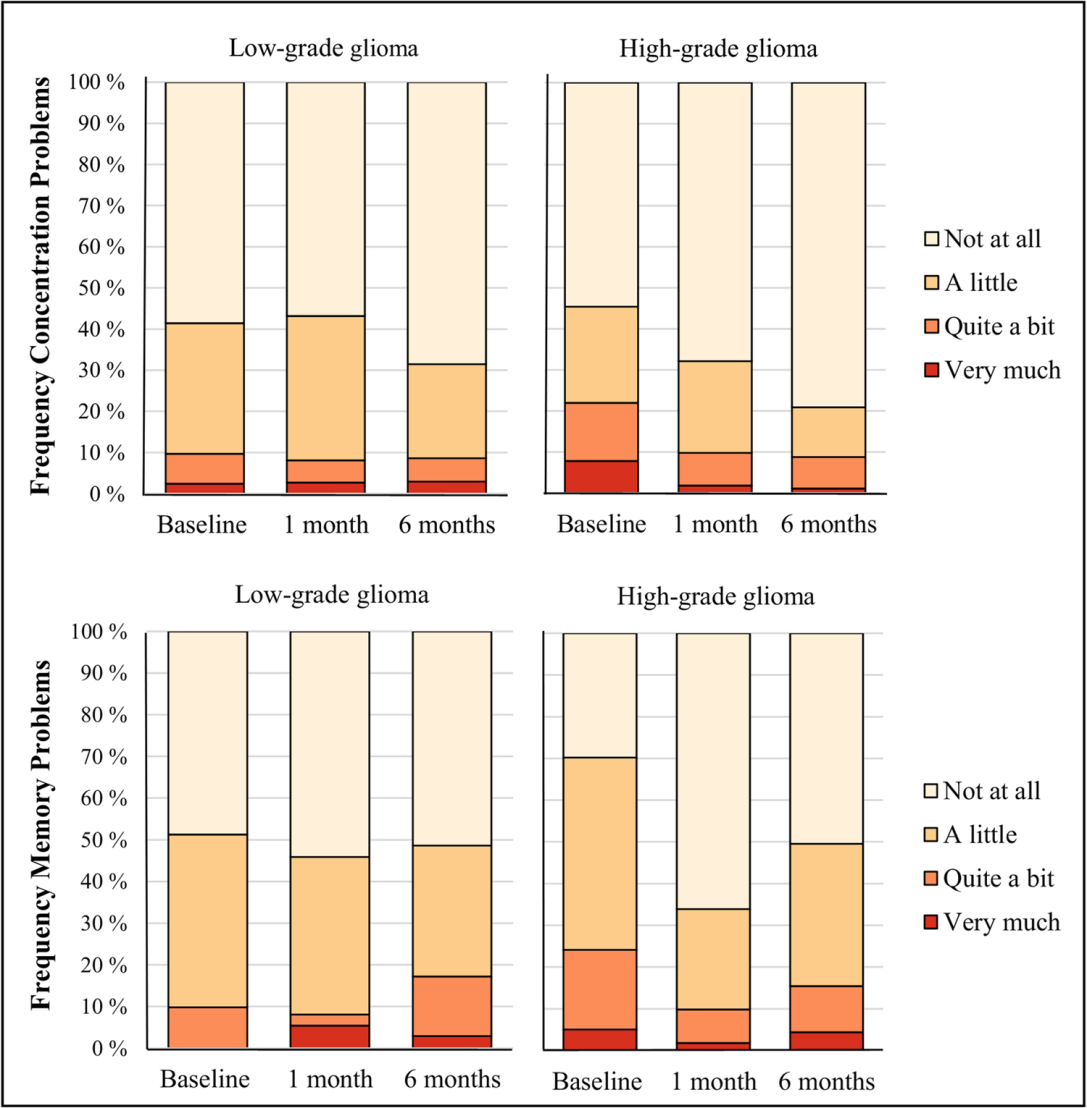
Distributions of symptom severity on outcome measures preoperatively and at 1 and 6 months postoperative follow-up

The distribution of symptom severity in LGG and HGG patients at each assessment is illustrated in Fig. 2. Among LGG patients, 41% reported concentration problems preoperatively, while 43% and 31% reported problems at 1 and 6 months of follow-up, respectively. Regarding memory, 51% of the LGG patients reported preoperative problems, and the proportions remained relatively stable at 1 and 6 months of follow-up. Among HGG patients, 45% reported concentration problems preoperatively, and the proportions with symptoms decreased to 32% and 21% at 1 and 6 months, respectively. Regarding memory, 70% of the HGG patients reported preoperative problems. The proportion decreased to 34% at 1 month before it increased to 50% at 6 months of follow-up.

### Individual changes in cognitive function

**Fig. 3:**
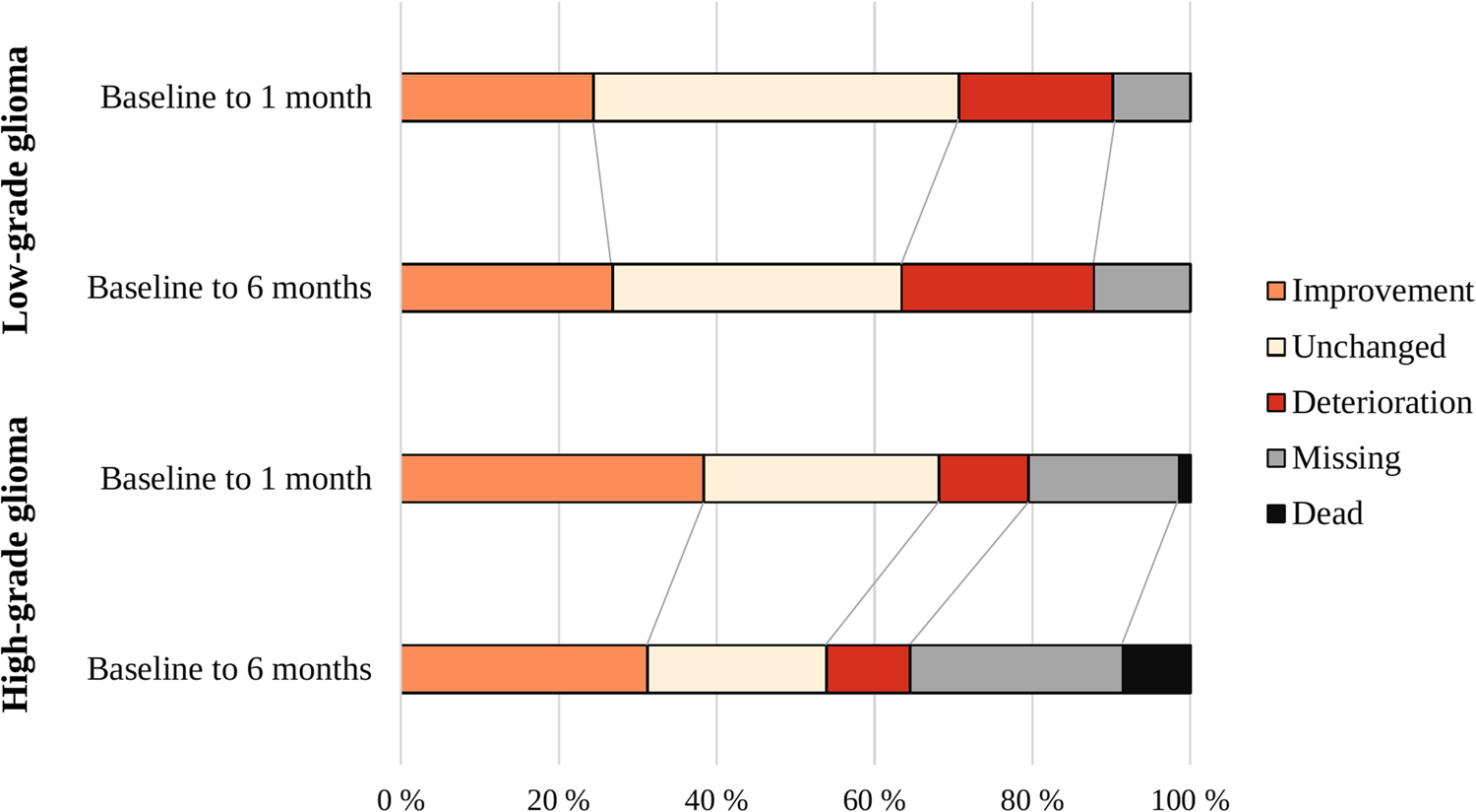
Clinically significant changes from preoperative to 1 and 6 months postoperative follow-up at an individual level

Figure 3 shows postoperative clinically significant changes in patient-reported cognitive function from preoperative to 1 and 6 months of follow-up at an individual level for LGG and HGG, with missing data and death as separate groups. In LGG patients, 24% reported improvement, 20% deteriorated, 46% were unchanged, and 10% had missing data at 1 month follow-up. From preoperative to 6 months, 27% reported improvement, and 24% reported cognitive deterioration.

In HGG patients, 38% reported improved cognitive function, 11% had deteriorated cognitive function, 30% were unchanged, 19% had missing data, and 2% were dead 1 month after surgery. However, more dynamics were seen from baseline to 6 months of follow-up in HGG patients. The proportion of patients with missing data increased to 27% and 9% died within 6 months of follow-up.

### Predictors associated with a change in cognitive function

Binomial logistic regression analyses were performed to explore potential predictors of changes in patient-reported cognitive function. Of factors reported in Table 1, histology, preoperative use of corticosteroids, preoperative tumor volume, and postoperative oncological treatment within 1 month were significantly associated with cognitive improvement from preoperative to 1 month of follow-up in the univariable analyses (Table 4). When including these variables in a multivariable model, corticosteroids and tumor volume remained significant independent predictors of cognitive improvement. Patients treated with preoperative corticosteroids had 3 times higher odds of reporting cognitive improvement 1 month after surgery than patients who did not use corticosteroids. For tumor volume, there was an increased odds of improvement with larger tumors in the groups up to 56.5 ml. The concordance index was 0.74, and the model explained 22.3% of the variance in cognitive improvement at 1 month. When exploring predictors associated with cognitive improvement from preoperative to 6 months of follow-up, preoperative KPS and preoperative use of corticosteroids were significantly associated in the univariable analyses. When including these in a multivariable model, no variables remained as significant independent predictors.

**Table 4:** Predictors for postoperative cognitive improvement at 1 and 6 months postoperative follow-up

	Univariable analyses		Multivariable analyses	
	OR (95% CI)	*p* value	OR (95% CI)	*p* value
Predictors for cognitive improvement at 1 month, *n* = 149				
High-grade glioma	2.51 (1.11, 5.68)	0.027*	0.53 (0.11, 2.48)	0.420
Preoperative corticosteroids	4.02 (1.91, 8.44)	< 0.001*	3.01 (1.10, 8.49)	0.032*
Preoperative tumor volume^a^		0.002*		0.023*
≤ 9.7 ml	Reference		Reference	
9.8–26.8 ml	4.22 (1.46, 12.18)	0.008*	3.62 (1.22, 10.79)	0.021*
28.5–56.5 ml	7.67 (2.60, 22.59)	< 0.001*	5.62 (1.84, 17.17)	0.002*
56.9–210.1 ml	5.74 (1.81, 18.20)	0.003*	3.98 (1.19, 13.28)	0.024*
Radiotherapy and/or chemotherapy within 1 month	2.64 (1.29, 5.42)	0.008*	1.95 (0.58, 6.56)	0.279
Predictors for cognitive improvement at 6 months, *n* = 126				
Preoperative KPS (continuous)	0.95 (0.92, 0.98)	0.003*	0.98 (0.93, 1.0)	0.079
Preoperative corticosteroids	2.90 (1.36, 6.17)	0.006*	1.80 (0.72, 4.51)	0.209

^*^indicates *p* ≤ 0.05

*CI*, confidence interval; *OR*, odds ratio; *KPS*, Karnofsky performance status

^*a*^*N* = 147 due to missing preoperative MRI

We also attempted to explore possible predictors associated with patient-reported cognitive worsening. However, no variables were significantly associated with cognitive worsening between preoperative and 1 and 6 months of follow-up in the univariable analyses.

## Discussion

In this prospective study, we assessed changes in patient-reported cognitive function following glioma surgery. At group level, lower preoperative cognitive function scores were seen in HGG than in LGG patients, and the scores were also lower in female patients than in males. While the HGG group reported better cognitive function scores at postoperative follow-ups, the LGG group reported stable scores. Perhaps surprisingly, the mean patient-reported cognitive function scores were comparable in patients with LGG and HGG at 1 and 6 months after surgery. However, missing data or death was more common in HGG at 6 months. At an individual level, both clinically significant improvements and deteriorations in cognitive function were frequently reported by both LGG and HGG patients. Preoperative use of corticosteroids and large tumor volume was associated with improvement at 1 month after surgery. However, we were unable to identify predictors for improvement at 6 months and worsening at 1 and 6 months. This study adds to the literature on the impact of glioma surgery on cognitive function, reflecting the patients’ perspective on the matter.

To our knowledge, this is the largest study to date that has assessed perioperative patient-reported cognitive function in diffuse glioma patients. In a small study of 22 patients with primary and recurrent grade I–IV glioma, cognitive change after surgery was assessed using the EORTC questionnaires at 7 days and 3 months postoperatively [45]. However, different study populations, assessment points, and no definition of clinically relevant change limit the comparison of results. Other glioma studies have mainly studied postoperative patient-reported cognitive function in oncological treatment studies [16, 32], where the outcome is found to be an important measure that correlates with objective testing [16]. The present study demonstrates the dynamics of patients’ perceived cognitive function in relation to glioma surgery. Thus, it underlines the importance of including patient-reported cognitive function as a construct in the neurosurgical setting, as recommended [3].

At group level, we found that patient-reported cognitive function scores and symptom severity improved in HGG patients following surgery while remaining stable in LGG patients. The majority of HGG are IDH-wild-type, and greater cognitive burden in IDH-wild-type gliomas has also been seen in neuropsychological studies [7]. There is suggested that rapid growth rate of IDH-wild-type gliomas tends to put more pressure on the surrounding structures than IDH-mutant gliomas [43]. Thus, extensive surgical resection may improve cognitive function by relieving the mass effect and edema. This could also explain that corticosteroids and large tumor volume were independently associated with postoperative improvement at 1 month after surgery in the present study. Interestingly, the odds of improvement were somewhat lower in the largest group of tumor volumes. It can be speculated whether the damage of the tumor is less reversible if the tumor becomes large enough. However, since few patients had very large tumors, this finding may be due to chance. It is also possible that side effects from corticosteroid treatment negatively affect subjective cognitive functional levels [18]. At individual level, HGG patients more often reported changes over time, perhaps influenced by oncological treatment, treatment responses, or tumor progression.

Patient-reported changes in cognitive function may also relate to factors other than the treatment itself, such as fatigue, pain, sleep disturbance, and the psychological effect of being diagnosed with a life-threatening disease [14, 15, 29, 31]. For example, anxiety, depression, and stress concerning the upcoming surgery could influence the patients’ perceived cognitive functioning [29]. After surgery, some patients will likely experience relief, while others may experience stress from the burden of having been diagnosed with cancer that needs further surveillance or oncological treatment. In accordance with other glioma studies, we found that females reported lower cognitive function scores than males, especially before surgery [2, 15]. In general, females seem to experience higher levels of psychological distress and are more willing to report their symptoms than males [26, 30].

The perceived cognitive function could also be attributed to self-expectations and environmental demands. LGG patients are often younger, less symptomatic prior to surgery, and more likely to resume work and family and social life [35]. Thus, they may have higher expectations to carry on with their usual activities after surgery. In contrast, HGG patients are more often elderly patients where only a minority return to work [38]. In addition, over time, patients will often adapt to their new situation, and a so-called response shift may be observed [37]. Although the effect of response shift is often small [20, 21], this may still have affected the cognitive function scores reported at 6 months of follow-up. Consequently, the complexity and possibly attributable factors to patient-reported cognitive function most likely partly explain the individual changes seen in our study.

Our findings indicate that the EORTC cognitive function subscale has the potential to capture changes in self-perceived cognitive function. This was also demonstrated in a recent study, where we found the prevalence of preoperative patient-reported cognitive impairment to be twice as high in diffuse glioma patients than in the general population [34]. Although objective testing remains the gold standard for structured cognitive evaluation, it may not always be available or feasible. As seen from clinical trials with neuropsychological endpoints, the ability to complete comprehensive tests may be problematic in those with poor prognoses [5, 6]. Thus, the study sample often inadequately reflects the patient population, and the external validity may be at risk [36]. Also, it may be practically difficult to perform objective testing after the patients are discharged from the hospital, especially in HGG patients. As a result, there is a relative lack of prospective data concerning long-term objective cognitive function in HGG. Patient-reported cognitive function may therefore be a practical tool to provide information about the patients’ cognitive health and disease status, especially in unselected glioma patients where extensive testing may be too burdensome.

Discrepancies between objective and subjective cognitive measures are known in the literature [8, 15, 31]. The patients may not be able to separate cognitive decline from fatigue or psychological distress. Further, some patients may be unaware of their cognitive impairment due to anosognosia, but according to a recently published study, many HGG patients are aware of their cognitive impairments after treatment [17]. If family and health care providers completed similar questionnaires on cognitive function, it could shed more light on possible self-awareness issues and inform the choice of compensation techniques and behavioral interventions. Nevertheless, glioma treatment aims to provide benefits for the patients, and therefore, the patient’s perspective of their cognitive function is of importance independent of objective test results. The advantage of self-reported cognitive measures is the ability to assess function in different real-world settings, unlike neuropsychological assessments performed in a more controlled setting where some patients may benefit from the quiet and structured test situation [10].

The strengths of this study are the prospective longitudinal design with preoperative assessment and the large population-based sample, increasing the generalizability of findings. Nevertheless, we cannot exclude an extent of selection bias due to preoperative non-inclusion and lost to follow-up. Missing data are common in glioma studies, especially in HGG patients. It is reasonable to assume that these patients would have reported cognitive deterioration if they had responded, and the cognitive function score in the HGG group may therefore be overestimated. However, we found little difference in patient-reported cognitive function scores between observed and estimated data. This may indicate a potential validity of the observed data in our material and strengthen our findings. Still, estimates are only based on data from patients that are alive. The EORTC cognitive subscale is a crude measure of patients’ perceived concentration and memory, and we could probably have detected more subtle impairments and differentiated better between degrees of impairment with a more detailed questionnaire. Moreover, since some patients received practical assistance to complete the questionnaire, we cannot exclude that this may have influenced their answers. At last, since the present study is based on prospectively included patients from 2011, IDH status was not available in all patients.

## Conclusion

Following glioma surgery, patients with HGG reported improvement of cognitive function scores postoperatively at group level, while LGG patients reported stable scores at all assessment points. Female patients reported lower cognitive function scores than males. At an individual level, both improvements and deteriorations in cognitive function were frequently reported in both LGG and HGG patients after surgery. Preoperative use of corticosteroids and large tumor volume was associated with improvement at 1 month after surgery. However, we were unable to identify predictors for improvement at 6 months and worsening at 1 and 6 months. This study adds to the literature on the impact of glioma surgery on cognitive function, reflecting the patients’ perspective on the matter.

## Data Availability

The dataset generated during and/or analyzed during the current study are not publicly available due to privacy concerns but are available from the corresponding author on reasonable request.

## Abbreviations

3D: Three-dimensional
CI: Confidence interval
EORTC: European Organisation for Research and Treatment of Cancer
FLAIR: Fluid-attenuated inversion recovery
HGG: High-grade glioma
IDH: Isocitrate dehydrogenase
KPS: Karnofsky performance status
LGG: Low-grade glioma
MCID: Minimal clinically important difference
MRI: Magnetic resonance imaging
OR: Odds ratio
RANO: Response Assessment in Neuro-Oncology
SD: Standard deviation

## References

[1] Aaronson NK, Ahmedzai S, Bergman B, Bullinger M, Cull A, Duez NJ, Filiberti A, Flechtner H, Fleishman SB, de Haes JC (1993) The European Organization for Research and Treatment of Cancer QLQ-C30: a quality-of-life instrument for use in international clinical trials in oncology. JNCI: J Natl Cancer Instit 85:365–376. 10.1093/jnci/85.5.365 10.1093/jnci/85.5.365 8433390

[2] Aaronson NK, Taphoorn MJ, Heimans JJ, Postma TJ, Gundy CM, Beute GN, Slotman BJ, Klein M (2011). Compromised health-related quality of life in patients with low-grade glioma. J Clin Oncol.

[3] Armstrong TS, Dirven L, Arons D, Bates A, Chang SM, Coens C, Espinasse C, Gilbert MR, Jenkinson D, Kluetz P (2020). Glioma patient-reported outcome assessment in clinical care and research: a Response Assessment in Neuro-Oncology collaborative report. Lancet Oncol.

[4] Boele FW, Zant M, Heine ECE, Aaronson NK, Taphoorn MJB, Reijneveld JC, Postma TJ, Heimans JJ, Klein M (2014). The association between cognitive functioning and health-related quality of life in low-grade glioma patients. Neuro-Oncology Practice.

[5] Brown PD, Ballman KV, Cerhan JH, Anderson SK, Carrero XW, Whitton AC, Greenspoon J, Parney IF, Laack NNI, Ashman JB, Bahary J-P, Hadjipanayis CG, Urbanic JJ, Barker FG, Farace E, Khuntia D, Giannini C, Buckner JC, Galanis E, Roberge D (2017). Postoperative stereotactic radiosurgery compared with whole brain radiotherapy for resected metastatic brain disease (NCCTG N107C/CEC·3): a multicentre, randomised, controlled, phase 3 trial. Lancet Oncol.

[6] Brown PD, Gondi V, Pugh S, Tome WA, Wefel JS, Armstrong TS, Bovi JA, Robinson C, Konski A, Khuntia D, Grosshans D, Benzinger TLS, Bruner D, Gilbert MR, Roberge D, Kundapur V, Devisetty K, Shah S, Usuki K, Anderson BM, Stea B, Yoon H, Li J, Laack NN, Kruser TJ, Chmura SJ, Shi W, Deshmukh S, Mehta MP, Kachnic LA, Oncology fN (2020). Hippocampal avoidance during whole-brain radiotherapy plus memantine for patients with brain metastases: phase III trial NRG oncology CC001. J Clin Oncol.

[7] Bunevicius A, Miller J, Parsons M (2020). Isocitrate dehydrogenase, patient-reported outcomes, and cognitive functioning of glioma patients: a systematic review. Curr Oncol Rep.

[8] Caramanna I, Bottomley A, Drijver AJ, Twisk J, van den Bent M, Idbaih A, Wick W, Pe M, Klein M, Reijneveld JC (2020). Objective neurocognitive functioning and neurocognitive complaints in patients with high-grade glioma: evidence of cognitive awareness from the European Organisation for Research and Treatment of Cancer brain tumour clinical trials. Eur J Cancer.

[9] Charlson ME, Pompei P, Ales KL, MacKenzie CR (1987). A new method of classifying prognostic comorbidity in longitudinal studies: development and validation. J Chronic Dis.

[10] Chaytor N, Schmitter-Edgecombe M (2003). The ecological validity of neuropsychological tests: a review of the literature on everyday cognitive skills. Neuropsychol Rev.

[11] Dallabona M, Sarubbo S, Merler S, Corsini F, Pulcrano G, Rozzanigo U, Barbareschi M, Chioffi F (2017). Impact of mass effect, tumor location, age, and surgery on the cognitive outcome of patients with high-grade gliomas: a longitudinal study. Neurooncol Pract.

[12] Fayers P, Aaronson NK, Bjordal K, Groenvold M, Curran D, Bottomley A (2001). EORTC QLQ-C30 Scoring manual.

[13] Fedorov A, Beichel R, Kalpathy-Cramer J, Finet J, Fillion-Robin J-C, Pujol S, Bauer C, Jennings D, Fennessy F, Sonka M, Buatti J, Aylward S, Miller JV, Pieper S, Kikinis R (2012). 3D Slicer as an image computing platform for the Quantitative Imaging Network. Magn Reson Imaging.

[14] Fox SW, Lyon D, Farace E (2007). Symptom clusters in patients with high-grade glioma. J Nurs Scholarsh.

[15] Gehring K, Taphoorn MJB, Sitskoorn MM, Aaronson NK (2015). Predictors of subjective versus objective cognitive functioning in patients with stable grades II and III glioma. Neuro-oncol Pract.

[16] Gilbert MR, Dignam JJ, Armstrong TS, Wefel JS, Blumenthal DT, Vogelbaum MA, Colman H, Chakravarti A, Pugh S, Won M, Jeraj R, Brown PD, Jaeckle KA, Schiff D, Stieber VW, Brachman DG, Werner-Wasik M, Tremont-Lukats IW, Sulman EP, Aldape KD, Curran WJ, Mehta MP (2014). A randomized trial of bevacizumab for newly diagnosed glioblastoma. N Engl J Med.

[17] Giovagnoli AR, Meneses RF, Paterlini C, Silvani A, Boiardi A (2021). Cognitive awareness after treatment for high-grade glioma. Clin Neurol Neurosurg.

[18] Habets EJJ, Kloet A, Walchenbach R, Vecht CJ, Klein M, Taphoorn MJB (2014). Tumour and surgery effects on cognitive functioning in high-grade glioma patients. Acta Neurochir (Wien).

[19] Ibañez FAL, Hem S, Ajler P, Vecchi E, Ciraolo C, Baccanelli M, Tramontano R, Knezevich F, Carrizo A (2011). A new classification of complications in neurosurgery. World Neurosurg.

[20] Ilie G, Bradfield J, Moodie L, Lawen T, Ilie A, Lawen Z, Blackman C, Gainer R, Rutledge RD (2019). The role of response-shift in studies assessing quality of life outcomes among cancer patients: a systematic review. Front Oncol.

[21] Jakola AS, Solheim O, Gulati S, Sagberg LM (2017). Is there a response shift in generic health-related quality of life 6 months after glioma surgery?. Acta Neurochir (Wien).

[22] Johnson DR, Sawyer AM, Meyers CA, O'Neill BP, Wefel JS (2012). Early measures of cognitive function predict survival in patients with newly diagnosed glioblastoma. Neuro Oncol.

[23] Louis DN, Ohgaki H, Wiestler OD, Cavenee WK, Burger PC, Jouvet A, Scheithauer BW, Kleihues P (2007). The 2007 WHO classification of tumours of the central nervous system. Acta Neuropathol.

[24] Louis DN, Perry A, Reifenberger G, von Deimling A, Figarella-Branger D, Cavenee WK, Ohgaki H, Wiestler OD, Kleihues P, Ellison DW (2016). The 2016 World Health Organization classification of tumors of the central nervous system: a summary. Acta Neuropathol.

[25] Maringwa J, Quinten C, King M, Ringash J, Osoba D, Coens C, Martinelli F, Reeve BB, Gotay C, Greimel E, Flechtner H, Cleeland CS, Schmucker-Von Koch J, Weis J, Van Den Bent MJ, Stupp R, Taphoorn MJ, Bottomley A (2011). Minimal clinically meaningful differences for the EORTC QLQ-C30 and EORTC QLQ-BN20 scales in brain cancer patients. Ann Oncol.

[26] Matud MP (2004). Gender differences in stress and coping styles. Pers Individ Dif.

[27] Mor V, Laliberte L, Morris JN, Wiemann M (1984). The Karnofsky performance status scale: an examination of its reliability and validity in a research setting. Cancer.

[28] Ng JCH, See AAQ, Ang TY, Tan LYR, Ang BT, King NKK (2019). Effects of surgery on neurocognitive function in patients with glioma: a meta-analysis of immediate post-operative and long-term follow-up neurocognitive outcomes. J Neurooncol.

[29] Nicol C, Ownsworth T, Cubis L, Nguyen W, Foote M, Pinkham MB (2019). Subjective cognitive functioning and associations with psychological distress in adult brain tumour survivors. J Cancer Surviv.

[30] Phillips DL, Segal BE (1969). Sexual status and psychiatric symptoms. Am Sociol Rev.

[31] Pranckeviciene A, Deltuva VP, Tamasauskas A, Bunevicius A (2017). Association between psychological distress, subjective cognitive complaints and objective neuropsychological functioning in brain tumor patients. Clin Neurol Neurosurg.

[32] Reijneveld JC, Taphoorn MJ, Coens C, Bromberg JE, Mason WP, Hoang-Xuan K, Ryan G, Hassel MB, Enting RH, Brandes AA (2016). Health-related quality of life in patients with high-risk low-grade glioma (EORTC 22033–26033): a randomised, open-label, phase 3 intergroup study. Lancet Oncol.

[33] Rijnen SJM, Sitskoorn MM, Gehring K (2019). Comment on: Effects of surgery on neurocognitive function in patients with glioma: a meta-analysis of immediate post-operative and long-term follow-up neurocognitive outcomes. J Neurooncol.

[34] Schei S, Solheim O, Salvesen Ø, Hjermstad MJ, Bouget D, Sagberg LM (2022). Pretreatment patient-reported cognitive function in patients with diffuse glioma. Acta Neurochir (Wien).

[35] Senft C, Behrens M, Lortz I, Wenger K, Filipski K, Seifert V, Forster M-T (2020). The ability to return to work: a patient-centered outcome parameter following glioma surgery. J Neurooncol.

[36] Skaga E, Skretteberg MA, Johannesen TB, Brandal P, Vik-Mo EO, Helseth E, Langmoen IA (2021). Real-world validity of randomized controlled phase III trials in newly diagnosed glioblastoma: to whom do the results of the trials apply?. Neuro-Oncology Advances.

[37] Sprangers MAG, Schwartz CE (1999). Integrating response shift into health-related quality of life research: a theoretical model. Soc Sci Med.

[38] Starnoni D, Berthiller J, Idriceanu T-M, Meyronet D, d’Hombres A, Ducray F, Guyotat J (2018). Returning to work after multimodal treatment in glioblastoma patients. Neurosurgical Focus FOC.

[39] Talacchi A, Santini B, Savazzi S, Gerosa M (2011). Cognitive effects of tumour and surgical treatment in glioma patients. J Neurooncol.

[40] Teixidor P, Gatignol P, Leroy M, Masuet-Aumatell C, Capelle L, Duffau H (2007). Assessment of verbal working memory before and after surgery for low-grade glioma. J Neurooncol.

[41] van Kessel E, Baumfalk AE, van Zandvoort MJ, Robe PA, Snijders TJ (2017). Tumor-related neurocognitive dysfunction in patients with diffuse glioma: a systematic review of neurocognitive functioning prior to anti-tumor treatment. J Neurooncol.

[42] van Kessel E, Huenges Wajer IMC, Ruis C, Seute T, Fonville S, De Vos FYFL, Verhoeff JJC, Robe PA, van Zandvoort MJE, Snijders TJ (2021). Cognitive impairments are independently associated with shorter survival in diffuse glioma patients. J Neurol.

[43] Wefel JS, Noll KR, Rao G, Cahill DP (2016). Neurocognitive function varies by IDH1 genetic mutation status in patients with malignant glioma prior to surgical resection. Neuro Oncol.

[44] Wen PY, Chang SM, Van den Bent MJ, Vogelbaum MA, Macdonald DR, Lee EQ (2017). Response assessment in neuro-oncology clinical trials. J Clin Oncol.

[45] Wolf J, Campos B, Bruckner T, Vogt L, Unterberg A, Ahmadi R (2016). Evaluation of neuropsychological outcome and “quality of life” after glioma surgery. Langenbecks Arch Surg.

